# Supplementary material for: In vitro assembly of plasmid DNA for direct cloning in Lactiplantibacillus plantarum WCSF1
Source: PLoS One. 2023 Feb 16;18(2):e0281625. doi: 10.1371/journal.pone.0281625 (PMC9934402; doi:10.1371/journal.pone.0281625)
Supplement: S1 File — (PDF) [file pone.0281625.s001.pdf]

JAN 31, 2023

OPEN ACCESS

In vitro assembly of plasmid DNA for direct cloning in Lactiplantibacillus plantarum WCSF1

Marc Blanch-Asensio[1], Sourik Dey[1], Shrikrishnan Sankaran[2]

[1]Bioprogrammable Materials Group, INM - Leibniz Institute for New Materials, Saarbrücken, Germany.;
[2]Bioprogrammable Materials Group, INM - Leibniz Institute for New Materials, Saarbrücken, Germany

Marc Blanch-Asensio

DOI: dx.doi.org/10.17504/protocols.io.ewov1o82olr2/v1

Protocol Citation: Marc Blanch-Asensio, Sourik Dey, Shrikrishnan Sankaran 2023. In vitro assembly of plasmid DNA for direct cloning in Lactiplantibacillus plantarum WCSF1. **protocols.io** https://dx.doi.org/10.17504/protocols.io.ewov1o82olr2/v1

License: This is an open access protocol distributed under the terms of the Creative Commons Attribution License, which permits unrestricted use, distribution, and reproduction in any medium, provided the original author and source are credited

Protocol status: Working
We use this protocol and it's working

Created: Sep 26, 2022

Last Modified: Jan 31, 2023

PROTOCOL integer ID: 70493

ABSTRACT

Protocol detailing a Gibson-assembly-based direct cloning method for *Lactiplatibacillus plantarum* WCFS1.

MOLECULAR CLONING

13h

1 VECTOR PCR

2h

Amplify the backbone sequence of interest. Always use a high-fidelity polymerase such as Q5 polymerase. PCR parameters:

- Use 10 ng of DNA as a template.
- Set the initial denaturation time to 1 min.
- Set the denaturation time during the cycles to 10 seconds.
- Extension: 30 sec/kb.
- Set the final extension time to 8 minutes.
- 28-cycle PCR.
- The volume of the reaction = 60 µl.

2 INSERT PCR

1h 30m

Amplify the sequence of interest using overhang primers to add the overhang sequences at the ends of the PCR product. The length of the overhang sequences depends on the commercial kit used for the Gibson Assembly. If the HiFi Assembly Master Mix from New England Biolabs (NEB) is used, an overhang of 20 base pairs is sufficient.

PCR parameters:

- Use 10 ng of DNA as a template.
- Set the initial denaturation time to 1 min.
- Set the denaturation time during the cycles to 10 seconds.
- Extension: 30 sec/kb.
- Set the final extension time to 8 minutes.
- 28-cycle PCR.
- The volume of the reaction = 80 µl.

3 GEL ELECTROPHORESIS

35m

1. Make a 1% agarose gel.

2. Run 5 µl of each PCR on an agarose gel electrophoresis to confirm amplification at the expected size. Run the gel for 30 min at 100 V.

4 DNA PURIFICATION

30m

1. Purify the PCR using the Wizard® SV Gel and PCR Clean-Up System from Promega following the protocol suggested by the manufacturer.

2. Measure the DNA concentration using a NanoDrop Microvolume UV-Vis Spectrophotometer.

5 GIBSON ASSEMBLY

40m

Assemble the vector and the insert using the HiFi Assembly Master Mix from New England Biolabs (NEB).

1. Define the amounts of vector and insert DNA required in this reaction following the protocol suggested by the manufacturer.

2. Mix the linear vector and insert DNA with overlapping DNA fragments with 10 µl of the HiFi DNA Assembly Master Mix.

3. Add Milli-Q water up to 25 µl.

4. Incubate the reaction at 50 °C for 30 minutes.

6 ASSEMBLED PRODUCT PCR

2h

Amplify the assembled product by PCR. Use a high-fidelity PCR such as Q5 polymerase.

1. Design primers that anneal to the insert and cover the whole assembled plasmid. An especially good set of primers is needed for this reaction. Properly check the primers with a primer design tool.

2. Use 5 µl of the HiFi Assembly reaction as a template for amplification. PCR parameters:

- Set the initial denaturation time to 2 min.
- Set the denaturation time during the cycles to 10 seconds.
- Extension: 30 sec/kb.
- Set the final extension time to 8 minutes.
- 23-cycle PCR.
- The volume of the reaction = 120 µl.

7 GEL ELECTROPHORESIS

35m

1. Make a 1% agarose gel.

2. Run 5 µl of each PCR on an agarose gel electrophoresis to confirm amplification at the expected size. Run the gel for 30 min at 100 V.

8 DNA PURIFICATION

30m

1. Purify the PCR using the Wizard® SV Gel and PCR Clean-Up System from Promega following the protocol suggested by the manufacturer.

2. Measure the DNA concentration using a NanoDrop Microvolume UV-Vis Spectrophotometer.

9 **QUICK BLUNTING REACTION (PHOSPHORYLATION)** 45m

Phosphorylate the 5´ends of the purified PCR products using the Quick Blunting Kit from NEB.

1. Mix 3500 ng of the purified DNA with 2.5 µl of the 10X Quick Blunting buffer and 1 µl of the Enzyme Mix.

2. Add Milli-Q water up to 25 µl.

3. Incubate the reaction first at 25 °C for 30 min.

4. Incubate the reaction at 70 °C for 10 min to inactivate the enzymes.

10 **LIGATION** 3h

Ligate the phosphorylated products using the T4 Ligase enzyme from NEB.

1. Mix 500 ng of the phosphorylated DNA (3.6 µl of the Quick Blunting reaction) with 1.5 µl of T4 Ligase and 2.5 µl of 10X T4 Ligase Buffer.

2. Add Milli-Q water up to 25 µl.

3. Incubate the reaction first at 25 °C for 2.5 hours.

4. Incubate the reaction at 70 °C for 10 min to inactivate the enzymes.

The number of ligation reactions differs based on the amount of DNA that is desired to be circularized.

11 **DNA PURIFICATION** 35m

1. Purify the PCR using the Wizard® SV Gel and PCR Clean-Up System from Promega following the protocol suggested by the manufacturer except for the elution step. Perform the elution three times (each time with 9 µl of Mili-Q water) in order to concentrate the ligated product.

2. Measure the DNA concentration using a NanoDrop Microvolume UV-Vis Spectrophotometer.

DNA is ready to be transformed.

LACTIPLANTIBACILLUS PLANTARUM WCFS1 ELECTROCOMP... 18h 15m

12 **INOCULATION WILD-TYPE BACTERIA** 5m

Inoculate Wild-type *L. plantarum* WCFS1 from glycerol stock in 5 mL of MRS media.

13 INCUBATION INITIAL CULTURE

14h

Incubate the bacterial culture overnight and at 37 °C with shaking (250 rpm).

14 SECONDARY CULTURE

5m

After approximately 16h, add 1 mL of the culture (OD600 = 2) to 20 mL of MRS media and 5 mL of 1% (w/v) glycine.

15 INCUBATION SECONDARY CULTURE

4h

Incubate this secondary culture for roughly 4 h at 37 °C and 250 rpm until OD600 reached 0.8.

16 WASHINGS

1h

1. Harvest the cells by centrifugation at 4000 rpm (3363 X g) for 12 min at 4°C.

2. Manually discard the supernatant.

3. Wash the pellet twice with 5 mL of ice-cold 10 mM MgCl2. Each time centrifuge cells at 4000 rpm (3363 X g) for 10 min at 4°C.

4. Wash the pellet twice (first with 5 mL and then with 1 mL) with ice-cold Suc/Gly solution (1 M sucrose and 10% (v/v) glycerol mixed in a 1:1 (v/v) ratio). Each time centrifuge cells at 4000 rpm (3363 X g) for 10 min at 4°C.

5. Manually discard the supernatant.

6. Resuspend the bacterial pellet in 450 µL of ice-cold Sac/Gly solution.

17 ALIQUOTING

5m

1. Make aliquotes of 60 uL each.

2. Immediately store at -80 °C the aliquots that are not going to be used.

3. Keep the aliquots that are going to be used for the transformation on ice.

18 ELECTROPORATION

15m

1. Mix the plasmid DNA (300 – 1200 ng) with 60 µl of freshly prepared electrocompetent cells.

2. Incubate the mixture on ice for 10 minutes.

3. Transfer the mixture to an ice-cold electroporation cuvette with a 2 mm gap (Bio-Rad Laboratories GmbH).

4. Insert the cuvette in the MicroPulser Electroporator (Bio-Rad Laboratories GmbH, Germany).

5. Apply a single pulse (5 ms) at 1.8 kV.

6. Add immediately 1 mL of room-temperature MRS media after the pulse.

7. Transfer the mixture into a 1.5 mL Eppendorf tube.

19 INCUBATION

3h

Incubate the bacteria at 37 °C and 250 rpm for 3 h to allow the expression of antibiotic resistance genes.

20 PLATING

5m

1. After the incubation, centrifuge the cells at 4000 rpm (3363 X g) for 5 min.

2. Discard the 800 µL of the supernatant.

3. Resuspend the remaining 200 µL by slow pipetting.

4. Plate the resuspended pellet on an MRS Agar plate supplemented with 10 µg/mL of Erythromycin.

21 INCUBATION

2d

Incubate the plate at 37 °C for 48 h for colonies to grow.

SEQUENCE VERIFICATION

17h 30m

22 COLONY PCR FOR SEQUENCE VERIFICATION

2h

1. Label the colonies to be screened on the plate.

2. For each colony, take half of the colony using a sterile pipette tip.

3. Scratch off the tip on a PCR tube.

4. Perform the PCR in this tube using the colony as the template for amplifying the gene of interest. Use primers that amplify the whole gene of interest. PCR parameters:

- Set the initial denaturation time to 10 minutes.
- Set the denaturation time during the cycles to 15 seconds.
- Extension: 30 sec/kb.
- Set the final extension time to 8 minutes.
- 28-cycle PCR
- Volume of the reaction = 100 µl.

Alternatively, the sequence verification PCR can also be done using the bacterial pellet instead of a colony. In this case:

1. Take 1 mL of an overnight culture (in a 1.5 mL Eppendorf tube) and centrifuge it at 4°C for 3 min at 8400 X g.

2. Manually discard the supernatant.

3. Scratched off the residual pellet fraction with a sterile pipette tip

4. Perform the PCR in this tube. Same PCR parameters as before.

23 GEL ELECTROPHORESIS 35m

1. Make a 1% agarose gel.

2. Run 5 µl of each PCR on an agarose gel electrophoresis to confirm amplification at the expected size. Run the gel for 30 min at 100 V.

24 DNA PURIFICATION 30m

1. Purify the PCR using the Wizard® SV Gel and PCR Clean-Up System from Promega following the protocol suggested by the manufacturer.

2. Measure the DNA concentration using a NanoDrop Microvolume UV-Vis Spectrophotometer.

25 SANGER SEQUENCING 15m

1. Send at least 1200 ng of purified DNA for sequencing. Higher amounts of DNA result in a better

reading.

2. Request an additional DNA purification step by the sequencing company (Eurofins Genomics). Additional Service: PCR Purification.

26 **INOCULATION** 5m

Take the remaining half of the colony and inoculate it into 5 mL of MRS media supplemented with 10 µg/mL of erythromycin.

27 **INCUBATION** 14h

Incubate the culture overnight at 37 °C with shaking (250 rpm).

28 **GLYCEROL STOCK** 5m

Mix 750 µl of the overnight culture with 250 µl of 60% glycerol. Store the tube at -80 °C.
