# Supplementary material for: In vitro assembly of plasmid DNA for direct cloning in Lactiplantibacillus plantarum WCSF1
Source: PLoS One. 2023 Feb 16;18(2):e0281625. doi: 10.1371/journal.pone.0281625 (PMC9934402; doi:10.1371/journal.pone.0281625)
Supplement: S2 File — (DOCX) [file pone.0281625.s002.docx]

**Supporting Information**

**In vitro assembly of plasmid DNA for direct cloning in *Lactiplantibacillus plantarum* WCSF1**

Marc Blanch Asensio,^1,†^ Sourik Dey,^1,†^ Shrikrishnan Sankaran*^1^

^1^ Bioprogrammable Materials, INM - Leibniz Institute for New Materials Campus D2 2, 66123 Saarbrücken, Germany

*E-mail: [Shrikrishnan.sankaran@leibniz-inm.de](mailto:Shrikrishnan.sankaran@leibniz-inm.de)

^†^ Authors contributed equally

| Primer name | Sequence |
| --- | --- |
| Vector mCherry fw | 5’-GAAGATAAATCCCATAAGGG-3’ |
| Vector mCherry rev | 5’-AAGAACTCTATTGAAGCCC-3’ |
| Insert mCherry fw | 5’-GGGGCTTCAATAGAGTTCTTGGTGATGTCGGCGATATAG-3’ |
| Insert mCherry rev | 5’-CCCTTATGGGATTTATCTTCGACTCGCACTGAGAGGAT-3’ |
| mCherry full amp fw | 5’-TTACAAGGCTAAGAAGCCAG-3’ |
| mCherry full amp rev | 5’-GTAGTCTTAACTTCAGCATCG-3’ |
| Vector elafin fw | 5’-AATAACTAGCATAACCCC-3’ |
| Vector elafin rev | 5’-CATGGTATATCTCCTTCTT-3’ |
| Insert elafin fw | 5’-TAAGAAGGAGATATACCATGATGCGTGCTTCATCATTC-3’ |
| Insert elafin rev | 5’-AAGGGGTTATGCTAGTTATTTTAGTGATGGTGATGGTG-3’ |
| Elafin full amp fw | 5’-CAAGAAGTGTTGTGAAGGT-3’ |
| Elafin full amp rev /  Elafin colony PCR rev | 5’-ATACCTGGACAATCAGTATCC-3’ |
| Elafin colony PCR fw | 5’-CAATGTTCCAAATGCGTG-3’ |
| Whole gene sequencing fw/  mCherry colony PCR fw | 5’-CGTTACTAAAGGGAATGGAG-3’ |
| Whole gene sequencing rev | 5’-AGTGGAACGAAAACTCAC-3’ |
| mCherry colony PCR rev | 5’- AGTTCATAACACGTTCCCAC -3’ |

**Table S1. List of primers used in this study.**

| **Gene** | **Sequence** |
| --- | --- |
| **mCherry** | **5’-**ATGGTTTCAAAGGGTGAAGAAGATAACATGGCTATCATCAAGGAATTCATGCGTTTCAAGGTTCACATGGAAGGTTCAGTTAACGGTCACGAATTCGAAATCGAAGGTGAAGGTGAAGGTCGTCCATACGAAGGTACTCAAACTGCTAAGTTAAAGGTTACTAAGGGTGGTCCATTACCATTCGCTTGGGATATCTTATCACCACAATTCATGTACGGTTCAAAGGCTTACGTTAAGCACCCAGCTGATATCCCAGATTACTTAAAGTTATCATTCCCAGAAGGTTTCAAGTGGGAACGTGTTATGAACTTCGAAGATGGTGGTGTTGTTACTGTTACTCAAGATTCATCATTACAAGATGGTGAATTCATCTACAAGGTTAAGTTACGTGGTACTAACTTCCCATCAGATGGTCCAGTTATGCAAAAGAAGACTATGGGTTGGGAAGCTTCATCAGAACGTATGTACCCAGAAGATGGTGCTTTAAAGGGTGAAATCAAGCAACGTTTAAAGTTAAAGGATGGTGGTCACTACGATGCTGAAGTTAAGACTACTTACAAGGCTAAGAAGCCAGTTCAATTACCAGGTGCTTACAACGTTAACATCAAGTTAGATATCACTTCACACAACGAAGATTACACTATCGTTGAACAATACGAACGTGCTGAAGGTCGTCACTCAACTGGTGGTATGGATGAATTATACAAGTAA **- 3'** |
| **Elafin** | **5’-** ATGCGTGCTTCATCATTCTTAATCGTTGTTGTTTTCTTAATCGCTGGTACTTTAGTTTTAGAAGCTGCTGTTACTGGTGTTCCAGTTAAGGGTCAAGATACTGTTAAGGGTCGTGTTCCATTCAACGGTCAAGATCCAGTTAAGGGTCAAGTTTCAGTTAAGGGTCAAGATAAGGTTAAGGCTCAAGAACCAGTTAAGGGTCCAGTTTCAACTAAGCCAGGTTCATGTCCAATCATCTTAATCCGTTGTGCTATGTTAAACCCACCAAACCGTTGTTTAAAGGATACTGATTGTCCAGGTATCAAGAAGTGTTGTGAAGGTTCATGTGGTATGGCTTGTTTCGTTCCACAAGGTGGTTCACATCACCATCACCATCACTAA **- 3'** |

**Table S2.** Genetic Sequences of the mCherry and Elafin coding segments used in this study.


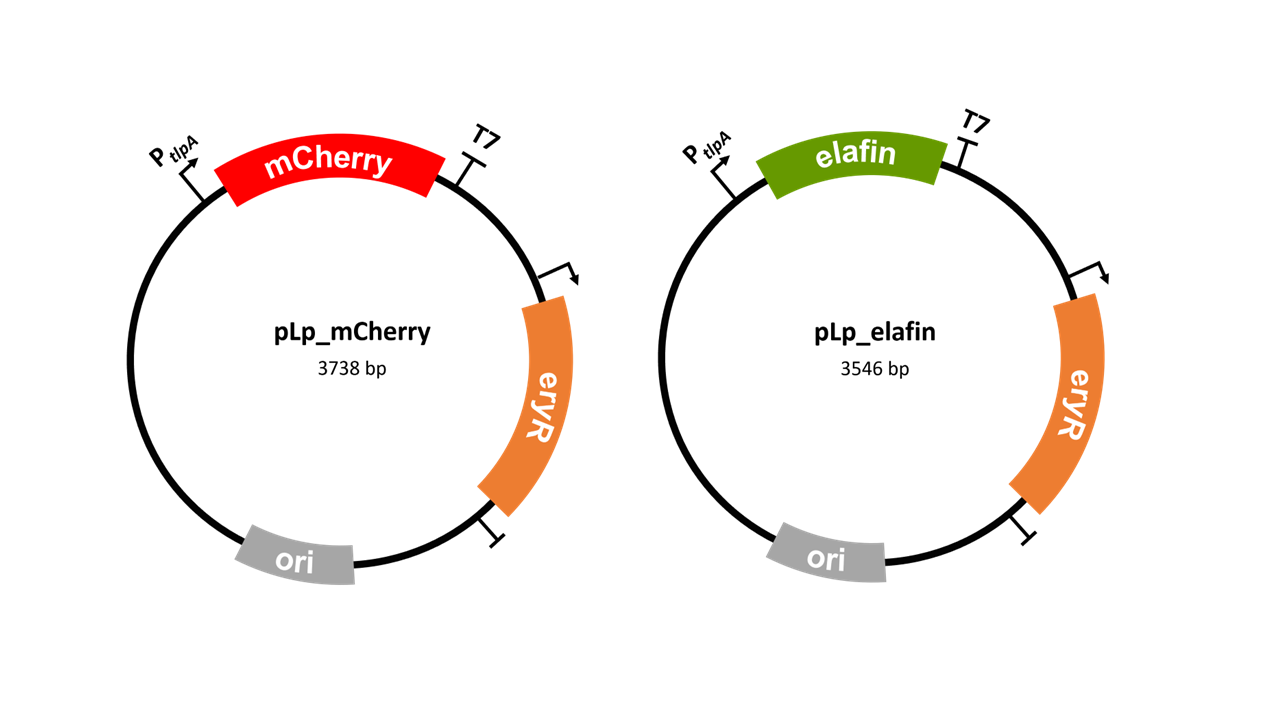


**Figure S1.** Schematic representation of the pLp_mCherry and pLp_elafin plasmids constructed in this study. The plasmid backbone is derived from the pLp3050sNuc plasmid (Mathiesen et al., 2009) and contains the P256 replicon and the erythromycin resistance gene cassette. The coding sequences of mCherry and elafin are under the strong P*_tlpA_* promoter. The T7 terminator is downstream of both the coding gene segments.


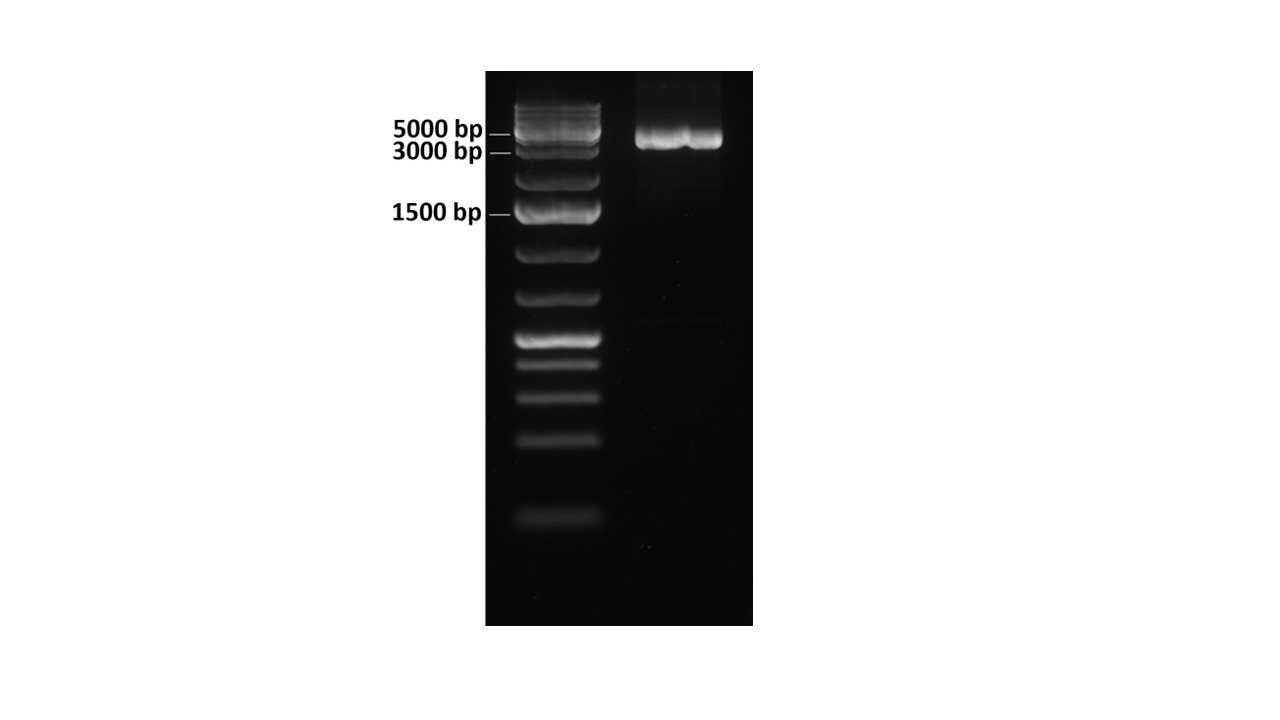


**Figure S2.** Agarose gel showing the PCR product (3728 bp) amplified from the assembled plasmid construct obtained after the Gibson Assembly Reaction. 1 kb Plus DNA ladder (ThermoFisher Scientific^TM^) was used for the reference.


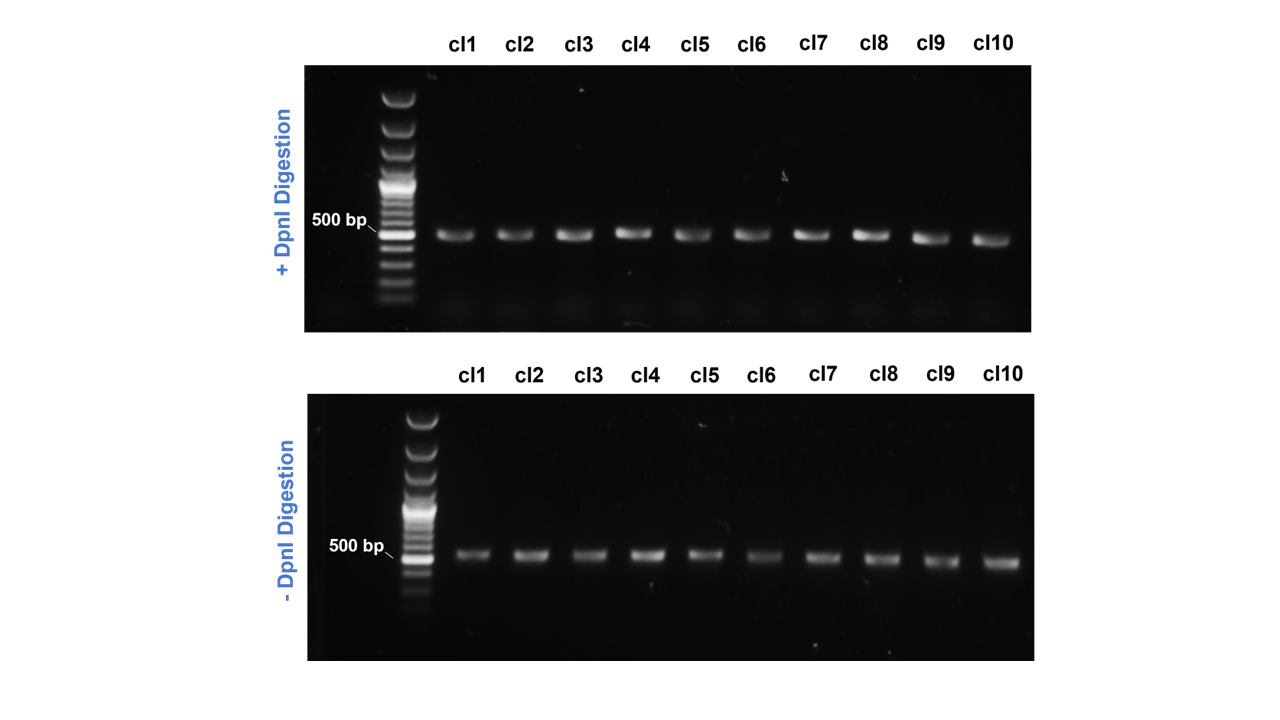


**Figure S3.** The agarose gel (top) is showing the colony PCR amplicon (558 bp) of 10 red *L. plantarum* colonies obtained through direct cloning with DpnI-treatment of the vector backbone. The agarose gel (bottom) is showing the colony PCR amplicon (558 bp) of 10 red *L. plantarum* colonies obtained through direct cloning without DpnI-treatment of the vector backbone. The PCR conditions were same for both the experimental setups. Generuler 100 bp Plus DNA Ladder (ThermoFisher Scientific^TM^) was used for the reference.


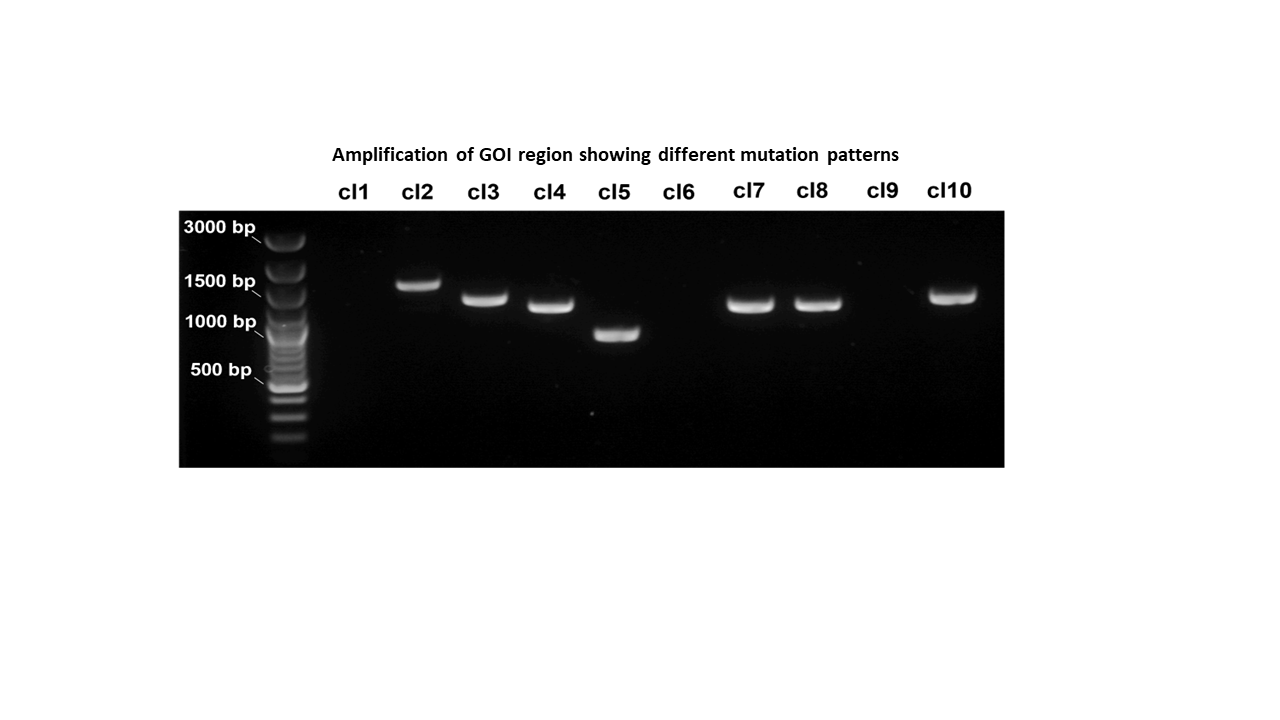


**Figure S4.** Agarose gel showing the colony PCR products corresponding to the GOI region from 10 non-red *L. plantarum* colonies obtained after direct cloning. The primer set used for amplification were the same as in **Figure 2C.** The different mutation patterns can be visualized for the respective colonies. Generuler 100 bp Plus DNA Ladder (ThermoFisher Scientific^TM^) was used for the reference.


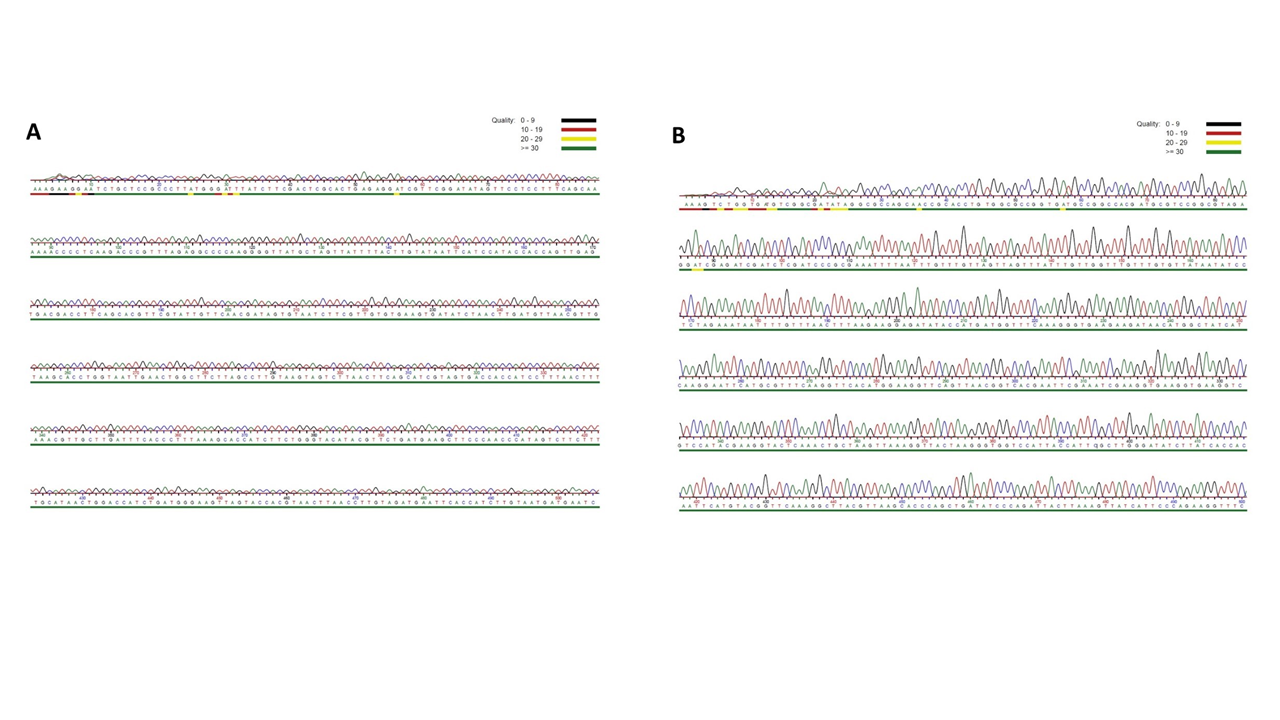


**Figure S5.** **Sequencing chromatograms.** A) Sequencing chromatogram without an initial purification step. B) Sequencing chromatogram with an initial purification step.


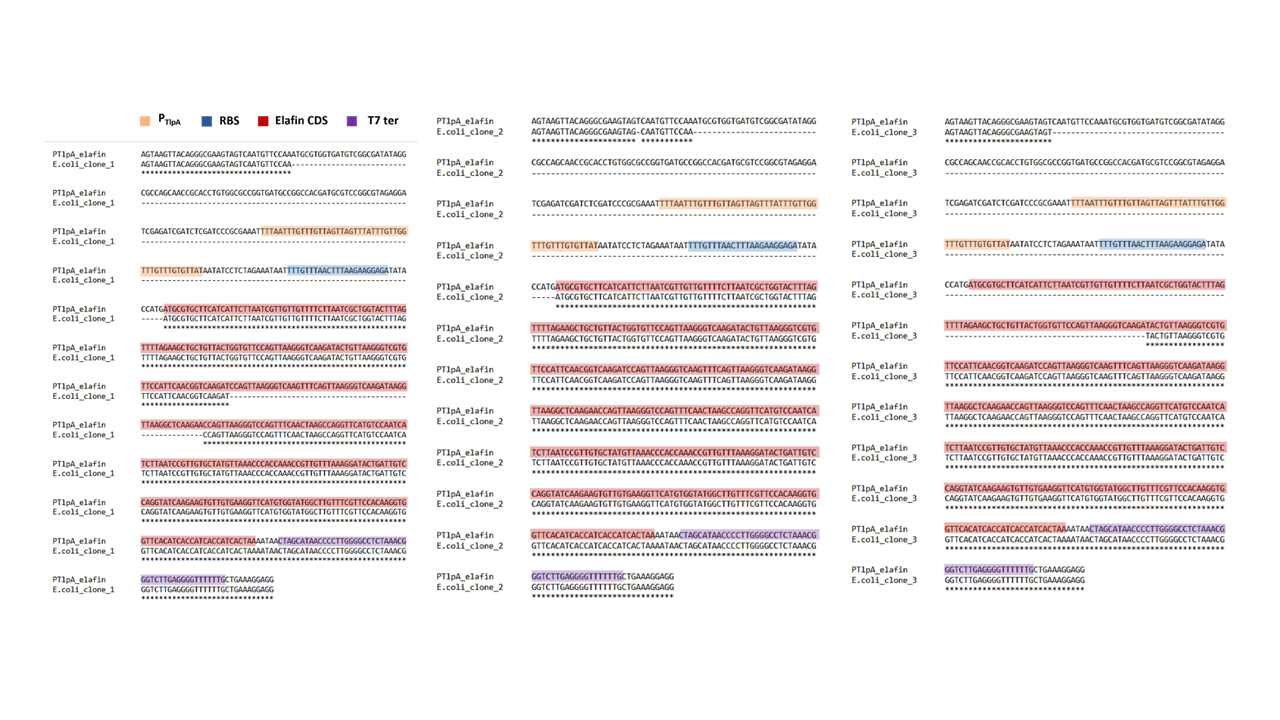


**Figure S6**. **Multiple sequence alignments of the sequencing results (MSA)**. The genetic sequence of the elafin gene was aligned to the gene sequences of the three pLp_elafin clones obtained through *E. coli* . All three clones showed deletions in the *P_TlpA_* promoter and RBS region. Clones 1 and 3 also showed additional deletions from the elafin coding sequence. The multiple sequence alignment tool (MUSCLE) was used for the comparative analysis of theSanger Sequencing results.
